# Study on the Compressive Properties of an Elastomeric Porous Cylinder Using 360° Three-Dimensional Digital Image Correlation System

**DOI:** 10.3390/ma16124301

**Published:** 2023-06-10

**Authors:** Wei Sun, Jie Zhao, Xin Li, Zhongda Xu, Zhenning Chen

**Affiliations:** College of Aerospace Engineering, Nanjing University of Aeronautics and Astronautics, Nanjing 210016, China; 18855481982@163.com (J.Z.); sx2201048@nuaa.edu.cn (X.L.); xuzhongda@nuaa.edu.cn (Z.X.); zhenning.chen@nuaa.edu.cn (Z.C.)

**Keywords:** 360° 3D DIC system, full surface strain measurement, coarse-fine matching method, elastomeric periodically porous cylinder, compressive properties

## Abstract

To study the compressive properties of an elastomeric porous cylinder, a 360° 3D digital image correlation (DIC) system is proposed. This compact vibration isolation table system captures different segments of the object from four different angles and fields of view, enabling a comprehensive measurement of the full surface of the object. To increase the stitching quality, a coarse–fine coordinate matching method is presented. First, a three-dimensional rigid body calibration auxiliary block is employed to track motion trajectory, which enables preliminary matching of four 3D DIC sub-systems. Subsequently, scattered speckle information characteristics guide fine matching. The accuracy of the 360° 3D DIC system is verified through a three-dimensional shape measurement conducted on a cylindrical shell, and the maximum relative error of the shell’s diameter is 0.52%. A thorough investigation of the 3D compressive displacements and strains exerted on the full surface of an elastomeric porous cylinder are investigated. The results demonstrate the robustness of the proposed 360° measuring system on calculating images with voids and indicate a negative Poisson’s ratio of periodically cylindrical porous structures.

## 1. Introduction

Nowadays, the demand for the complete 3D surface measurement of objects is increasing with the rise of 3D DIC technology. However, traditional systems using two cameras can only capture certain local surfaces or the front of an object [1]. To address this limitation, multi-camera systems are preferred due to their wide field of view and accurate measurement results [2,3]. Ruihai Xin et al. measured and analyzed the 360-degree full-field deformation of the cylindrical shell structure by using a multi-DIC system; images taken from various angles could be arranged in the same coordinate system with the rigid point cloud, whose reference points are attached on the head of the UTM [4]. Weifu Sun et al. combined the DIC technique with non-destructive probing procedures to design and build a 360° three-dimensional (3D) DIC system to reveal the displacement fields of the cylindrical shells. The DIC technique is used to detect and localize the dominating imperfection location, and automatic reconstructions of point clouds are obtained by a single system in the global coordinate system through the DLT method [5]. Multi-camera systems can shoot different areas of the object from multiple angles and fields of view and unify local coordinate systems using calibration and image stitching to accomplish 3D profile reconstruction, displacement, and strain measurement of the entire object surface [6,7].

There are typically four methods available for conducting full surface measurements on objects with significant curvature. The first method involves rotating a camera completely around the object and capturing images of it at the same angle after each rotation [8]. Yapeng Guo et al. proposed a displacement field calculation framework for large-scale structures based on computer vision with physical constraints and using a single camera set on the automatic rotating device to obtain high-resolution images of large-scale structures [9]. The second method involves placing the object on a turntable and rotating it by a fixed angle relative to a single fixed camera or binocular camera system before capturing images [10]. The third method involves using specialized optical components with reflection and refraction functions to obtain a panoramic view of the object [11,12]. Kaiyu Zhu et al. proposed a mirror-assisted single-camera panoramic DIC method for panoramic/dual-surface profile and deformation measurement of regular-sized test objects [13]. Xiao Zhou et al. used the prism assembly and M-array coding method to achieve the fast and high-precision measurement of a large field of view by multi-camera collaboration [14]. The fourth method involves surrounding the object with a multi-camera setup in which all cameras capture images synchronously while maintaining a fixed spatial position relative to the object [15,16,17]. Junrui Li introduced a multi-camera DIC system which uses four cameras to measure the whole-field thickness strain in detail [16]. Siyuan Fang et al. presented a new R-value measurement method based on the use of two stereo DIC systems to measure thickness strain at any point on a sheet metal surface [18]. Rui Zhu et al. developed a multi-camera array system for full-field modal measurements [19]. Although the first and second methods are suitable for measuring static or quasi-static deformation, the object cannot move and it requires manual intervention for capturing images. The third and fourth methods can facilitate dynamic 3D measurement along 360° circumferential directions, but their devices are bulky and may not be portable. Meeting spatial requirements within narrow spaces poses a significant challenge. Whereas the advantages of utilizing a camera with a wide field of view for accurate measurements across a broad range of applications are evident, its prohibitive cost and occupied physical footprint further compound the difficulties. The adoption of multi-camera systems exacerbates this issue, necessitating unification and data stitching between disparate systems while also ensuring synchronization of each individual camera’s shooting process.

To address these challenges, this paper proposes a compact eight-camera 3D DIC system for the complete surface measurement of objects with large curvature. By optimizing existing eight-camera arrangement methods and proposing a vertical stereoscopic measurement approach, the hardware trigger mode based on synchronization signal generation resolves camera synchronization in quasi-static experimental states. A method for system stitching is proposed that includes a coarse–fine coordinate system matching method and the design of a 3D rigid body calibration auxiliary block [16]. Multiple systems are preliminarily matched by tracking the motion trajectory of the calibration block [20]. Fine matching between systems is then carried out using the same speckle information in the overlapping fields of view [21]. The global coordinate system is unified after completing the fine matching. The consistency of data and measurement accuracy of each system is verified through a three-dimensional shape measurement conducted on a cylindrical shell. The holes on the whole surface of the porous cylindrical periodic structure specimen are segmented and filtered using a morphological image preprocessing method, which allows for the successful realization of the dynamic compression experimental measurement of the whole surface of the specimen. The content of this paper is arranged as follows. Section 2 describes coarse–fine coordinate system matching method. Section 3 reports on two experiments: one aims to verify the accuracy of the 360° 3D DIC system, whereas the other is for studying the compressive properties of an elastomeric porous cylinder. Section 4 concludes this paper.

## 2. Coarse–Fine Coordinate System Matching Method

To satisfy the needs of industrial metrology, it is common practice to consolidate multiple cameras into an array to expand the visual field of coverage. Accordingly, the precise calibration and alignment of the interconnections between every camera system within a multi-camera configuration are deemed of utmost significance in the realm of multi-camera 3D DIC methodology.

### 2.1. Multi-Camera Calibration Method Based on Rigid Body Motion

The crux of a multi-camera measurement system resides in the seamless amalgamation of the data collected from diverse subsystems. This study utilizes a multi-camera calibration technique founded on the principles of rigid body motion. The aforementioned approach predominantly employs a rigid body calibration block that is replete with speckles printed on its surface to calibrate inter-subsystem relationships. Notably, the dimensions of the rigid body calibration block are commensurate with those of the specimen. In comparison with the conventional multi-camera matching method predicated on overlapping fields of view, the multi-camera matching technique centered on rigid body motion offers several advantages. These include: (1) the ability to leverage the entire camera field of view with no necessity for overlapping fields of view between camera systems and (2) the simplicity and practicability of the calibration system’s fabrication and operation.

The presented approach consists of three principal steps.

Step 1: The dot calibration plate is utilized for the calibration of the internal and external parameters of each camera system in the setup [22].

Step 2: A series of rigid body motions are conducted on the object of interest, which exists within different visual systems (Sys1, Sys2, Sys3, Sys4). During this step, each system captures a distinct surface of the rigid body as it undergoes controlled motions. Notably, the rigid body’s rigidity prohibits any deformation during movement, thereby ensuring that the various surface calibrators remain in unified world coordinates. As a result, the calculated trajectories of the motion vectors of the different surfaces remain consistent. By collecting motion pictures at multiple intervals, the positional changes and motion trajectory of the rigid object in space can be estimated and accurately tracked. The methodology thus offers a robust and precise means for the motion tracking of rigid objects [20].

**Figure 1:**
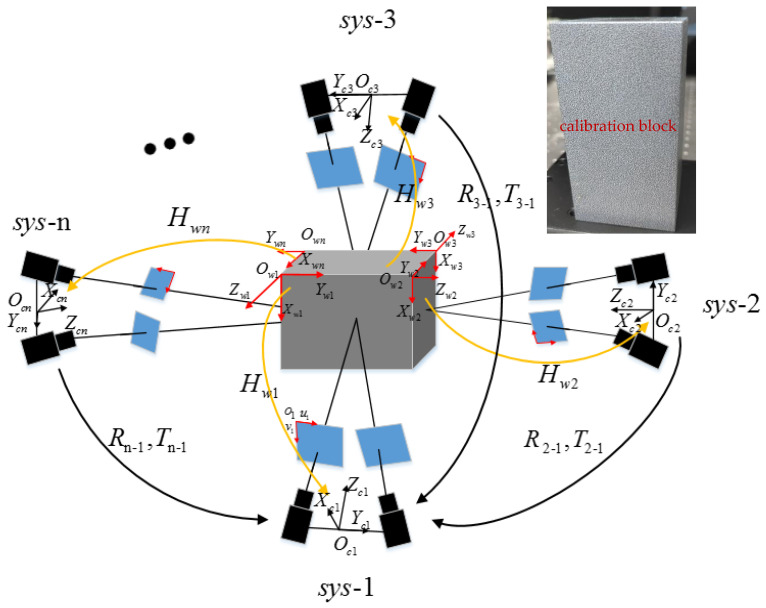
Schematic of geometric transformations within a multi-camera system.

A schematic of the geometric transformations in a multi-camera system and the calibration block is shown in Figure 1. In accordance with the fundamental principle of 3D calibration and matching, the interrelation between the 2D image coordinate system and the 3D world coordinate system may be established as follows:

(1) $$Q_{w}^{i}=H_{wi}Q_{c}^{i}$$

where $Q_{c}^{i}$ denotes the pixel coordinate of the center of a sub-region that has been selected from the pixel coordinate system i of the left camera, $Q_{w}^{i}$ refers to the corresponding point coordinate in the world coordinate system i of the rigid body, $H_{wi}(i=1,2,\ldots ,n)$ is the projection transformation matrix pertaining to the camera pixel coordinate system and the world coordinate system within the subsystem i. Following the calibration procedures outlined in step 1, a set of projection transformation matrices ($H_{wi}$) from each camera system to the corresponding calibration system is derived. By analyzing the speckle patterns printed on the surface of the rigid body, we obtain a robust estimate of the underlying motion of the object under its respective system. This information is then used to reconstruct a series of point cloud data representing the motion of the rigid body plane.

Step 3: The binocular stereovision technique is utilized to reconstruct the 3D world coordinates of each surface point of the rigid body object, while simultaneously solving the vector motion trajectory of each surface in relation to the center of gravity. Additionally, the iterative closest point (ICP) [23] algorithm is employed in conjunction with the least square method to obtain the rotation matrix and translation matrix between distinct world coordinate systems. This enables the calibration of the spatial position relationship of the camera systems to be accomplished and the seamless integration of the local coordinate system into a world coordinate system.

### 2.2. Fine-Matching Method Based on Speckle Information

**Figure 2:**
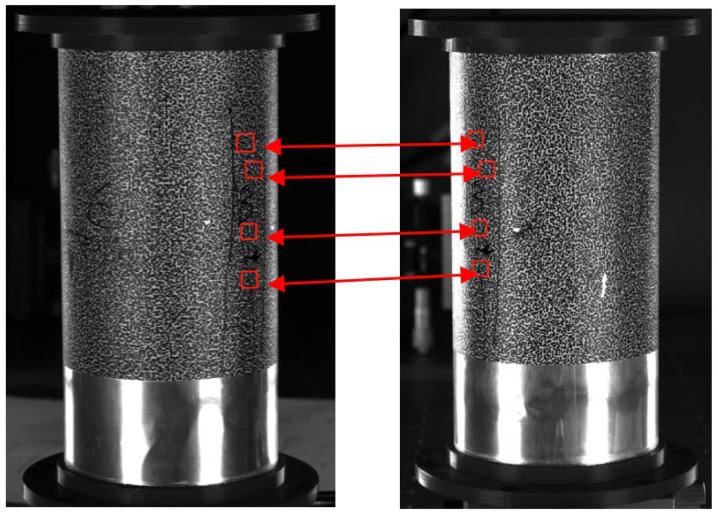
Schematic of the process of matching scattered spots.

The multi-system registration method based on rigid body motion can calibrate and unify multiple systems at the same time without matching between two systems, which reduces the complexity of operation and avoids error transfer. However, this method which is affected by the flatness of the rigid body surface, external vibration and illumination, is a simple multi-system coarse registration method. As shown in Figure 2, through using speckle information carriers that have the same overlapping area and obtaining accurate feature identification points through speckle image matching, the spatial correspondence that is based on these feature identification points can be established and the rotation matrix and the translation matrix between different world coordinate systems can be solved by the quaternion method.

The sample is captured by a multi-camera system from two different angles with partial overlapping areas. The system then calculates a 3D data point set for the corresponding points in the two areas, labeled as *P* and *Q*. For a subset of *N* points $\left\{m_{i}|m_{i}\in P,i=1,2,..,N\right\}$ selected from the *P* data point set, there is an equivalent subset containing *N* points $\left\{{m^{\prime }}_{i}|{m^{\prime }}_{i}\in Q,i=1,2,..,N\right\}$ in the *Q* data point set that corresponds to every point in *P*. To match the overlapping area, the system seeks to find the coordinate conversion relationship *R* and *T*, where *R* represents the rotation matrix and *T* represents the translation matrix, between the 3D data points in the two coordinate systems. Typically, *R* and *T* can be obtained by minimizing Equation (2):

(2) $$J={\sum_{i=1}^{N}\left\|{m^{\prime }}_{i}-(Rm_{i}+T)\right\|}^{2}$$

Finally, all measurement points are converted and unified within the same world coordinate system to create a complete data model. This process eliminates the need for a separate calibration procedure and does not rely on a large overlap between cameras. The final stitching result is based on the rough stitching approach from the multi-system calibration method using rigid body motion.

## 3. Experiment and Analysis

### 3.1. Experimental System

The experimental setup comprised eight Basler industrial cameras (model acA4112-30um), each possessing a resolution of 4096 × 3008 pixels and capable of capturing 300 frames per second. A number of 35mm Kowa low distortion fixed-focus lenses were employed uniformly across all cameras. In order to optimize the field of view and ensure maximal utility between the cameras, a rational configuration for the eight-camera system was determined [24].

**Figure 3:**
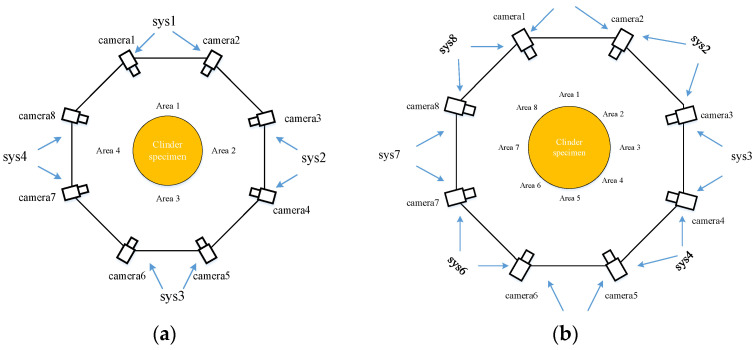
Two traditional camera arrangements. (**a**) Sequential combination; (**b**) Adjacent coupling combination.

Although theoretically, the effective field of view for a single camera on a cylinder is 180°, corresponding to half of the cylindrical surface, practical considerations such as depth of field and illumination lower the effective field of view to a range of 120°–150°. The approach depicted in Figure 3a involves combining two cameras in succession and requires the same number of calibrations and 3D reconstructions as the arrangement method proposed in this paper. However, this approach results in a decrease in the overlapping field of view between the two cameras in each system, potentially posing challenges in achieving complete 360° full surface stitching and measurement. Conversely, the arrangement method illustrated in Figure 3b enables the acquisition of numerous overlapping areas yet entails up to eight calibrations and 3D reconstructions which is twice the number required for the proposed approach and could lead to data confusion.

**Figure 4:**
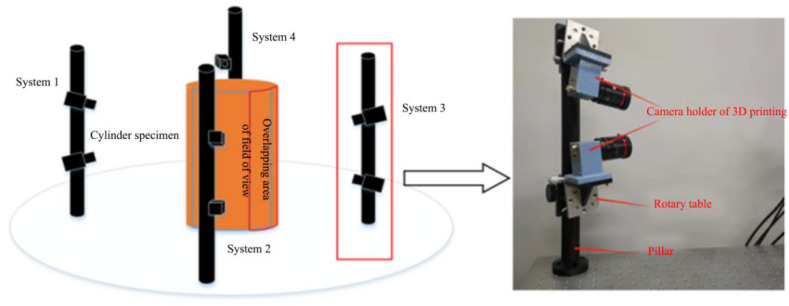
Eight-camera system arranged vertically.

Based on the aforementioned arrangements, a straightforward and efficient configuration for an eight-camera measurement system is proposed in this paper. The eight cameras are divided into four pairs, each pair forming a stereo DIC measurement system. The front, left, right, and rear surfaces of the cylinder specimen are then photographed by the four measuring systems. Figure 4 illustrates the experimental arrangement. The two cameras in each system are arranged vertically and fixed on a pillar. A rotary table is located at the bottom of the camera to adjust the pitch angle. By vertically arranging the cameras, the overlapping field of view of the two systems can be equivalent to the field of view of a single camera by adjusting the pitch angle. This configuration simplifies the calibration process and fulfills the requirement for full surface measurement. Moreover, the overlapping field of view between the two systems is significantly increased when compared with the previous arrangement.

The experiment utilized hardware control to synchronize the cameras [25]. The synchronization signal controller was connected to the camera’s Power I/O signal trigger interface to control synchronous image acquisition. The camera’s USB 3.0 interface was responsible for transmitting the collected data and powering the camera. Due to the limited interface type and development cycle, the RIGOL DG1022U synchronous signal controller was used. However, during the actual data transmission for the eight cameras, the acquisition rate had to be reduced due to limited computer channels (only two), slow hard disk reading rates, and a maximum USB 3.0 transmission bandwidth of 500 MB/s. As a result, the acquisition rate of the eight cameras simultaneously was only 8 fps based on testing. The maximal temporal discrepancy computed, which amounts to 17 milliseconds, satisfies the prerequisites for conducting a quasi-static experiment.

**Figure 5:**
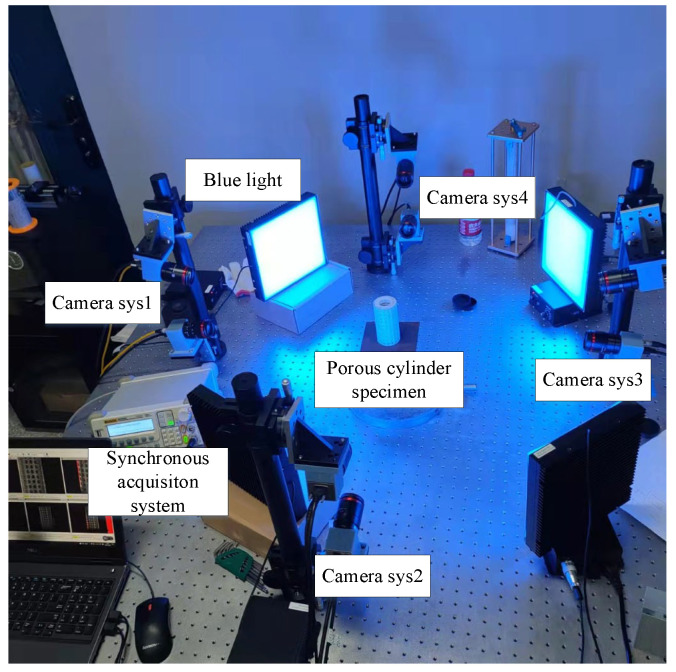
Experimental diagram of cylindrical displacement measurement.

Figure 5 presents the experimental setup employed for the cylinder specimen, which was designed as follows: four binocular camera systems were arranged vertically encompassing the specimen, whereas four blue light sources were symmetrically positioned between the aforementioned camera systems in order to guarantee an even distribution of illumination. Every camera’s Power I/O signal trigger interface was connected to the synchronization signal controller and the USB 3.0 interface was connected to a computer. The specimen was placed on a cross bracing and located at the center of the four camera systems. Notably, the entire surface of the specimen was adorned with speckle patterns that had been randomly printed. The printer model employed in this paper was UJF-A3HG, whose manufacturer is Mimaki, in Tokyo, Japan.

### 3.2. Preprocessing on the Full Surface of an Elastomeric Porous Cylinder Using the Filling Method in Morphology

**Figure 6:**
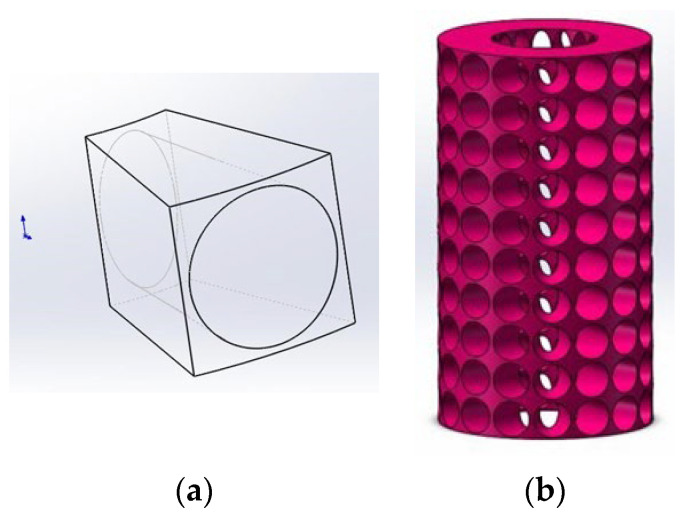
Model of elastomeric porous metamaterial. (**a**) Single cell; (**b**) Porous periodic structure.

The full surface measurement of porous cylindrical periodic structures is achieved through the implementation of an eight-camera DIC system. The first step involved constructing a 3D model of the porous cylinder, as depicted in Figure 6a. The model was composed of periodically arranged cells, where each cell was a fan-shaped structure with an external diameter *R_o_* and an internal diameter of *R_i_* = 1/2 *R_o_*. The angle of the fan-shaped region was determined by the number of openings around the cylinder, denoted as *N_c_*, and was given by *α* = 2π/*N_c_*. The height of a single cell, denoted as *L_u_*, was calculated as *L_u_* =*αR_o_*. To facilitate measurement using the DIC system, holes of different sizes were opened on the front and back surfaces of the solid single cell, as illustrated in Figure 6a. The hole on the external surface was circular, with a radius of *R_p_* = *αR_o_*/2. On the other hand, the hole on the internal surface was ellipsoid, with the long axis and short axis sizes designated as *A_p_* = *αR_o_*/2 and *B_p_* = *α_i_*/2, respectively. Upon determination of the dimensions of a singular unit cell, a cylindrical structure that contained 16 perforations spanning across ten layers was fabricated through the application of SolidWorks three-dimensional modeling software, specifically utilizing the loft-cut and circular pattern operations as illustrated in Figure 6b.

In order to generate optimized digital speckle on the surface of a porous cylindrical periodic structure, one must overcome the challenge of speckle printing within the porous cylinder, which can lead to the creation of various chaotic backgrounds inside the cylindrical holes. This presents a significant impediment to the calculation of digital image correlation as the area inside the hole must also be included for accurate data analysis, resulting in disordered point cloud data and failure to accurately analyze buckling data.

**Figure 7:**
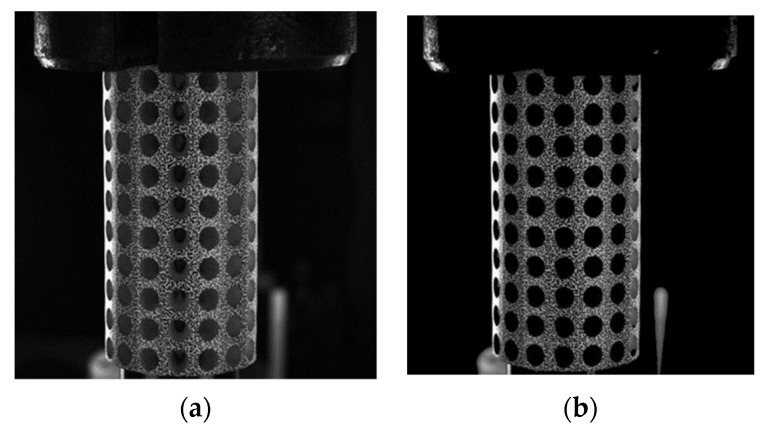
The images of the porous cylindrical specimen before and after processing. (**a**) Image before processing; (**b**) Image after processing.

To address this issue, we propose a novel morphological image preprocessing approach that includes the preprocessing of non-speckle regions inside the holes and the extraction and zeroing of invalid regions inside the holes. The proposed procedure involves a set of specific operations, namely adaptive threshold banalization, multi-resistant noise morphology operations, and convolution with the original image. With this preprocessing method, we can ensure that these areas are excluded from relevant calculations and eliminate the chaotic background inside the holes, thereby enhancing the accuracy and efficiency of analysis. The effectiveness of our approach is demonstrated through a comparison of specimen images before and after processing, as illustrated in Figure 7.

### 3.3. Experimental Results

#### 3.3.1. The 360° 3D Shape Measurement of a Cylinder Shell

Initially, the internal and external parameters of each subsystem are acquired via a calibration process. Subsequently, speckle patterns are printed on the surface of a fabricated cubic calibration plate. The relationship matrix between the four systems is then determined using a multi-view registration method founded on rigid body motions. This is obtained by calculating the speckle patterns. Systematic matching is subsequently executed. By adopting system one as the principal coordinate system, the transformation matrices of the other three coordinate systems relative to system one were computed.

**Figure 8:**
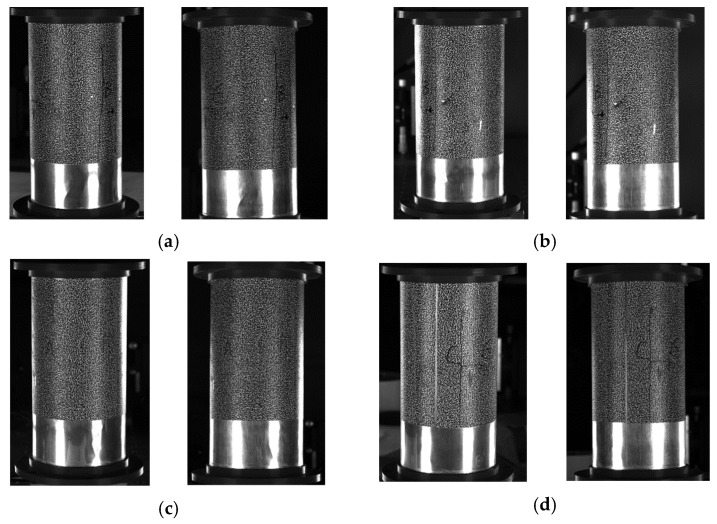
Images captured by four subsystems. (**a**) System 1; (**b**) System 2; (**c**) System 3; (**d**) System 4.

Within the scope of the captured reference images presented in Figure 8, a total of eight images were obtained. Among these, two cameras positioned vertically between each of the systems were incorporated to maximize the resolution of the cameras. The utilization of such cameras ensures an ample coverage area for the speckle patterns that overlap between adjoining systems, facilitating the acquisition of abundant data points that can be utilized for the precise matching of speckles in subsequent analyses.

**Figure 9:**
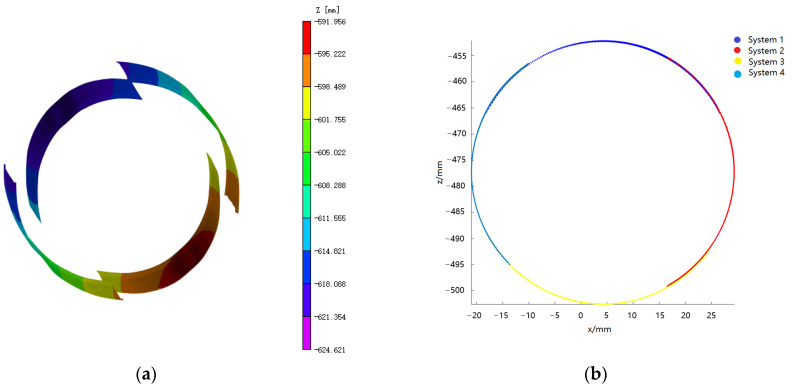
Three-dimensional shape of the cylindrical shell after stitching. (**a**) Coarse stitching; (**b**) Fine stitching.

The present study involves the selection of the region of interest (ROI) from the four subsystems delineated in Figure 8. The 3D coordinates of the ROI are then calculated using the conventional 3D DIC system. Furthermore, the transformed coordinates of the 3D data points in each of the four subsystems are analyzed after transformation, based on the established relationship between them through the registration method of rigid body motion. The resulting 3D shape of the cylindrical specimen is obtained by stitching the acquired data together, as depicted in Figure 9a.

After utilizing the rigid body motion-based multi-view registration approach to merge and register the four systems, a well-formed continuous topography pattern can typically be obtained. Due to the fact that the world coordinates of speckles located in the overlapping region across different systems are equivalent, the transformation matrix between the two systems can be computed via the extraction of coordinate points in distinct systems. Notably, both system 2 and system 4 possess fields of view that overlap with system 1, enabling them to be transformed into system 1 based on the aforementioned transformation matrix. Conversely, for system 3, which is located opposite to system 1, no overlapping area exists between the two systems, precluding system 3 from being directly transformed into system 1. Thus, system 3 must be converted into system 2 or system 4, which can then be transformed into system 1. However, it should be noted that this methodology may yield secondary error transmission between systems [16]. The 3D shape of the spliced surface is depicted in Figure 9b, which exhibits the seamless continuity of the cylindrical specimen and the elimination of dislocation at the joint.

Based on the data obtained from cylindrical surfaces, surface fitting can be performed to determine the curvature of each system. The resulting curvature data and relative errors are presented in Table 1. Analysis of this data reveals that the calculated curvatures for the four parts are highly consistent, indicating excellent accuracy of both the calibration data and the stitching algorithm. Moreover, through fitting the cylindrical specimen, the average value of the cylindrical diameter at the reference stage was determined as 50.32 mm, which was then compared with the reference diameter (50.06 mm) measured with a vernier caliper. The absolute error of measurement was calculated as 0.26 mm and the relative error was determined to be 0.52%, achieving the necessary precision for practical measurement purposes.

#### 3.3.2. Full Surface Compressive Test on an Elastomeric Porous Cylinder

**Figure 10:**
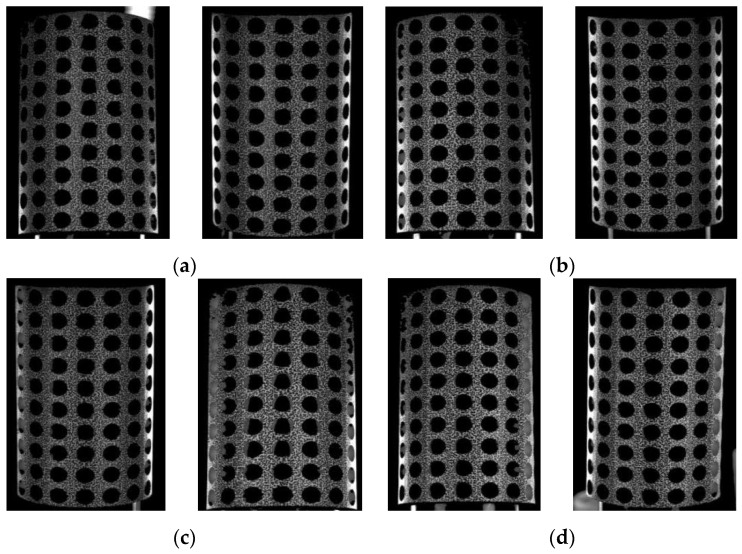
Images captured from four subsystems after preprocessing. (**a**) System 1; (**b**) System 2; (**c**) System 3; (**d**) System 4.

After performing batch morphological preprocessing on the specimen images acquired from the four systems, a set of images is presented in Figure 10. The chaotic background inside the holes has been effectively eliminated. By incorporating the multi-system coarse–fine registration approach, the data from all four systems are integrated and amalgamated to obtain a comprehensive displacement and strain map of the porous cylindrical periodic structure. Prior to image preprocessing, it is imperative to select a larger sub-region when choosing sub-regions for computation to account for the presence of gray data in the pore, which ensures a smooth and uninterrupted progress for the pertinent calculations. Following preprocessing, all data within the holes has been determined to be uniformly zero. Consequently, there is no requirement to account for the impact of these vacant regions on the computation of the correlation function when selecting sub-regions for analysis. Moreover, this selection process substantially reduces the size of the sub-region, diminishing from 89 × 89 pixels to a mere 35 × 35 pixels. This approach enabled expedited computational processing and augmented the amount of data analyzed while maintaining consistency between matched regions.

**Figure 11:**
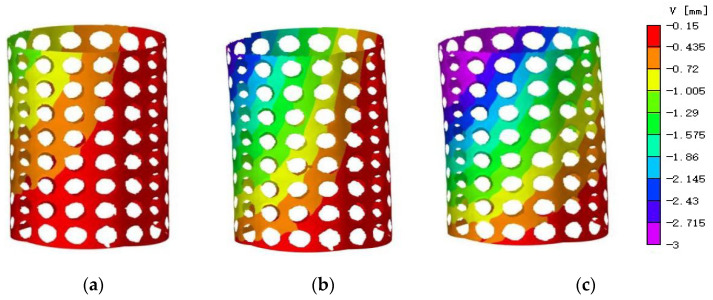
Vertical displacement map under different loads. (**a**) 20 N; (**b**) 40 N; (**c**) 70 N.

**Figure 12:**
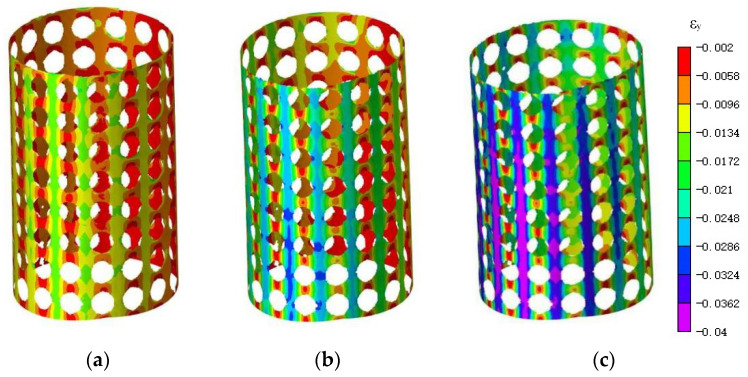
Vertical strain map under different loads. (**a**) 20 N; (**b**) 40 N; (**c**) 70 N.

During the compression process, Figure 11 and Figure 12 illustrate the trend of vertical displacement and strain variation of the specimen under the respective loads of 20 N, 40 N, and 70 N. The non-uniform distribution of axial displacement in the compression direction and the asymmetric axial displacement field are clearly depicted in the displacement map and attributed to the specimen’s eccentric loading in the compression test. The strain map, after undergoing image preprocessing, exhibits improved data continuity and discerns a conspicuous concentration of strain at the periphery of the aperture.

**Figure 13:**
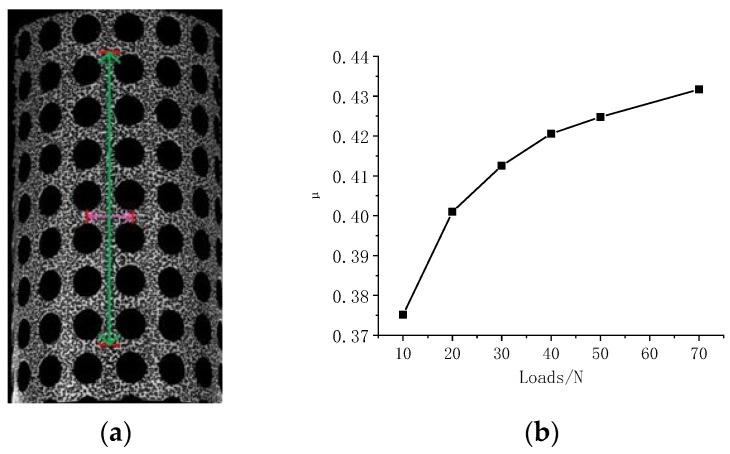

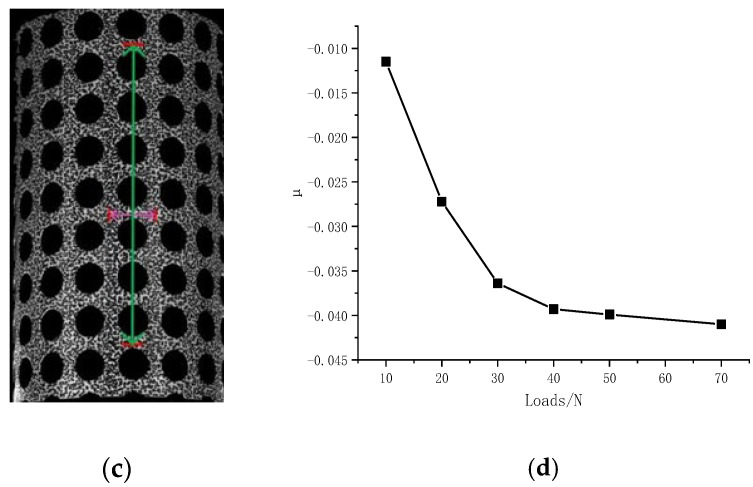
Poisson’s ratio curves. (**a**) Solid strip; (**b**) Poisson’s ratio curve for a solid strip; (**c**) Hollow strip; (**d**) Poisson’s ratio curve for a hollow strip.

In this study, Poisson’s ratios for both solid and hollow strips are derived by utilizing the methodology illustrated in Figure 13a,c. Specifically, two distinct distances located along the circumferential and vertical directions are selected to evaluate the axial and circumferential variation of the two line segments during deformation. The resulting Poisson’s ratios of these segments are presented in Figure 13b,d. The findings suggest a substantial Poisson’s ratio in the solid strip data, whereas a negative Poisson’s ratio is observed in the hollow strip data. As the load increases, the Poisson’s ratio of both the solid and hollow strips shows a nonlinear growth trend. The cylinder used is made of rubber material, and the Poisson’s ratio of rubber is between 0.4 and 0.5. Therefore, in Figure 13b, the Poisson’s ratio approximates to the material’s characteristics, whereas in Figure 13d, it is a characteristic of the pore structure. However, these exceptional structure and material properties demonstrate the vast potential application of the porous cylindrical periodic structure. In addition, the data derived from the aforementioned methodology affirm the dependability of the eight-camera DIC measurement approach.

Due to the fact that all of our experimental equipment is installed on the vibration isolation table and the compression testing machine cannot be used, we can only load the specimen with counterpoises, whose weight is insufficient to measure the compressive strength [26]. However, we can measure the distribution of the 360° strain field during compression, which shows the non-uniformity of strain distribution from various perspectives.

## 4. Conclusions

In this paper, a compact 360° eight-camera 3D DIC system was designed and developed for the purpose of enabling the complete surface measurement of cylinder specimens featuring significant curvature. This system has certain advantages in measuring objects with complex geometric shapes, nonuniform strain, or significant curvature, such as the cases for eccentric load, structural asymmetry, defects, or material’s anisotropy.

Through the application of the aforementioned method, discrepancies arising from stitching are corrected and multifarious data fusion and stitching protocols are then implemented. In our experimental analysis, a cylinder shell was selected and the fitting curvature error calculated via four separate systems was demonstrated to be less than 0.24%. With integration of a specifically tailored morphological image preprocessing method, we successfully isolated, segmented, and filtered the pores present across the entirety of a porous cylindrical periodic structural specimen. Finally, compression testing under incremental loads was conducted across its entire surface area, conclusively showcasing the large Poisson’s ratio and negative Poisson’s ratio exhibited by the elastomeric porous cylinder. As the load increases, the Poisson’s ratio of both solid and hollow strips shows a nonlinear growth trend. By analyzing the 360° strain field distribution map, it is apparent that the eccentric load is observable and the degree of eccentricity can be calculated.

Multi-camera systems have a wide range of applications but there are also corresponding technical issues. On one hand, multi-camera systems require high-performance hardware equipment, including a computer that can perform high-speed processing, high-speed data transmission lines, a hard disk that can be read at high speed, and so on. If high-speed cameras are used, the data volume will increase exponentially. Further experimental measurement research on high-speed dynamics can be conducted, such as impact problems and high-speed vibration problems. In addition, when measuring a cylindrical shell with a longitudinal corrugated pipe and the height of the ripples exceeds a certain threshold, the acquisition of a clear image can prove to be an arduous task. This difficulty arises primarily due to the limitations of uniform illumination and the depth of field of the imaging system.

## Figures and Tables

**Table 1:** Curvature and error of each system.

	Standard	System 1	System 2	System 3	System 4
Curvature (1/mm)	0.02	0.019976	0.019952	0.019979	0.020013
Relative error	0	0.12%	0.24%	0.11%	0.065%

## Data Availability

Not applicable.
